# Supplementary material for: YAP1 mediates survival of ALK-rearranged lung cancer cells treated with alectinib via pro-apoptotic protein regulation
Source: Nat Commun. 2020 Jan 3;11:74. doi: 10.1038/s41467-019-13771-5 (PMC6941996; doi:10.1038/s41467-019-13771-5)
Supplement: Supplementary file 1 — Supplementary Information [file 41467_2019_13771_MOESM1_ESM.pdf]

Supplementary information

Table of contents

Supplementary Table 1-3

Supplementary Figure 1-9

Supplementary Methods

Supplementary References

*Supplementary Data 1.2.3 are provided as separate excel files.

Supplementary Data 1: The whole result of proteome analysis

Supplementary Data 2: Gene ontology (GO) analysis and KEGG pathway analysis on proteome

Supplementary Data 3: Results of whole exome sequencing on H2228ARY and H2228

Supplementary Tables

Supplementary Table 1: Characteristics of 13 patients with ALK-rearranged lung cancer

No	Stage	IHC/FISH ALK	PS	PFS (days)	Smoking status	Pre-treatment (mm^3)	First evaluation (mm^3)		Best response (mm^3)		Disease progression (mm^3)	
1	IVa	+/+	1	941 (continuing)	ex-smoker	20209	839	PR	438	PR	N/A	
2	IVb	+/+	1	224 (continuing)	ex-smoker	15252	3429	PR	858	PR	N/A	
3	IVa	+/+	2	240 (PD)	non-smoker	11113	5339	PR	2940	PR	22016	PD
4	IVa	+/+	0	1381 (continuing)	ex-smoker	29501	6679	PR	6679	PR	N/A	
5	Rec.	+/+	1	1176 (continuing)	ex-smoker	1614	80	CR	31	CR	N/A	
6	IIIb	+/+	1	818 (continuing)	ex-smoker	3056	2213	PR	196	PR	N/A	
7	IVb	+/+	1	148 (continuing)	non-smoker	445	155	PR	16	CR	N/A	
8	IVa	+/+	0	1408 (continuing)	non-smoker	26036	2750	PR	48	CR	N/A	
9	IVb	+/+	1	163 (PD)	ex-smoker	29501	6679	PR	6679	PR	46817	PD
10	IVa	+/+	0	239 (PD)	non-smoker	24419	11960	PR	756	PR	4447	PD
11	IVb	+/+	1	327 (continuing)	non-smoker	6738	6520	SD	5625	SD	N/A	
12	IVa	+/+	1	315 (continuing)	non-smoker	28800	3600	PR	63	CR	N/A	
13	IVa	+/+	0	1065 (continuing)	non-smoker	7228	3912	PR	63	CR	N/A	

IHC: Immunohistochemistry, FISH: fluorescent *in situ* hybridization, ALK: anaplastic lymphoma kinase, PS: performance status, PFS: progression-free survival, Rec: postoperative recurrence, PR: partial response, CR: complete remission, SD: stable disease, PD: progression disease, N/A: Not applicable.

Supplementary Table 2. Antibody list

**Antibody list**			
**Primary antibodies**	**Dilution rate**	**Manufacture**	**Clone/Cat. No.**
YAP1	1:500 (IB)<br>1:500 (IHC, *in vitro*)<br>1:100 (IHC, xenograft)	Santa Cruz Biotechnology	sc-101199
YAP1	1:50 (ChIP)	Cell Signaling Technology	D8H1X
Phospho-YAP (Ser127)	1:1000 (IB)	Cell Signaling Technology	D9W2I
Mcl-1	1:1000 (IB)	Cell Signaling Technology	D35A5
GAPDH	1:2000 (IB)	ThermoFisher Scientific	GA1R
Bcl-xL	1:1000 (IB)	Cell Signaling Technology	54H6
Cleaved PARP	1:1000 (IB)	Cell Signaling Technology	D64E10
Vinculin	1:2000 (IB)	Abcam	ab18058
Phospho-ALK(Tyr1604)	1:1000 (IB)	Cell Signaling Technology	#3341
ALK	1:1000 (IB)	Cell Signaling Technology	C26G7
Cleaved Caspase-3 (Asp175)	1:1000 (IB)	Cell Signaling Technology	#9661
Lats1	1:1000 (IB)	Cell Signaling Technology	C66B5
Phospho-Lats1 (Ser909)	1:1000 (IB)	Cell Signaling Technology	#9157
Pan-Akt	1:1000 (IB)	Cell Signaling Technology	C67E7
Phospho-Akt (Ser473)	1:2000 (IB)	Cell Signaling Technology	D9E

**Secondary antibodies**	**Dilution rate**	**Manufacture**	**Clone/Cat. No.**
Anti-mouse IgG, HRP-linked antibody	1:2000 (IB)	Cell Signaling Technology	#7076
Anti-rabbit IgG, HRP-linked antibody	1:2000 (IB)	Cell Signaling Technology	#7074
Anti-mouse/Rabbit IgG (H+L), Biotinylated	1:300 (IHC, xenograft)	Vector Lab	BA-1400

IB: immunoblotting, IHC: immunohistochemistry, ChIP: Chromatin immunoprecipitation.

Supplementary Table 3. Primer sequences for the quantitative polymerase chain reaction

Quantitative Reverse Transcription PCR (qRT-PCR) Primers		
**Amplicon**	**Forward (5'-->3')**	**Reverse (5'-->3')**
YAP1	CCCGACAGGCCAGTACTGAT	CAGAGAAGCTGGAGAGGAATGAG
TAZ	GGCTGGGAGATGACCTTCAC	CTGAGTGGGGTGGTTCTGCT
MCL1	CCAAGGCATGCTTCGGAAA	TCACAATCCTGCCCCAGTTT
BCL2	ATGTGTGTGGAGAGCGTCAACC	TGAGCAGAGTCTTCAGAGACAGCC
BIRC5	TCCGGTTGCGCTTTCCT	TCTTCTTATTGTTGGTTTCCTTTGC
BCLW	GGGGTAAACTGGGGTCGCATT	ACCAGCGGTTGAAGCGTTC
BCLXL	GGTATTGGTGAGTCGGATCG	AAGAGTGAGCCCAGCAGAAC
NANOG	AGTAAAGGCTGGGGTAGGTAGG	GAACTCTCCAACATCCTGAACC
POU5F1	GGTATTCAGCCAAACGACCA	CACACTCGGACCACATCCTT
BCLXL	GGTATTGGTGAGTCGGATCG	AAGAGTGAGCCCAGCAGAAC
NANOG	AGTAAAGGCTGGGGTAGGTAGG	GAACTCTCCAACATCCTGAACC
POU5F1	GGTATTCAGCCAAACGACCA	CACACTCGGACCACATCCTT
SOX2	CCTCCGGGACATGATCAG	TTCTCCCCCCTCCAGTTC
CD133	AAGCATTGGCATCTTCTATGG	AAGCACAGAGGGTCATTGAGA
ALDH1A1	TGTTAGCTGATGCCGACTTG	TTCTTAGCCCGCTCAACACT
GAPDH	AGGGCTGCTTTTAACTCTGGT	CCCCACTTGATTTTGGAGGGA
ABCB1	CGTGGTTGGAAGCTAACCCT	TGCTGCCAAGACCTCTTCAG
**ChIP-qPCR Primers**		
**Amplicon**	**Forward (5'-->3')**	**Reverse (5'-->3')**
MCL1-A	AACCACATTGTCAGGCCTCT	GTGGGGCAGAAACAAATCAC
MCL1-B	TCCCAACCTCTTAACTCCAAAA	TGAGTGGCAAGGATAGAGCA
BCLXL-A	GTCCACTGGTGCTTTCGATT	GGGAGAGAAAGAGCTTCAGGA
BCLXL-B	AGCGAGACCCTGTCAAGAAA	TCCCTGCCTCTTCGTATTCA

Supplementary Figure 1

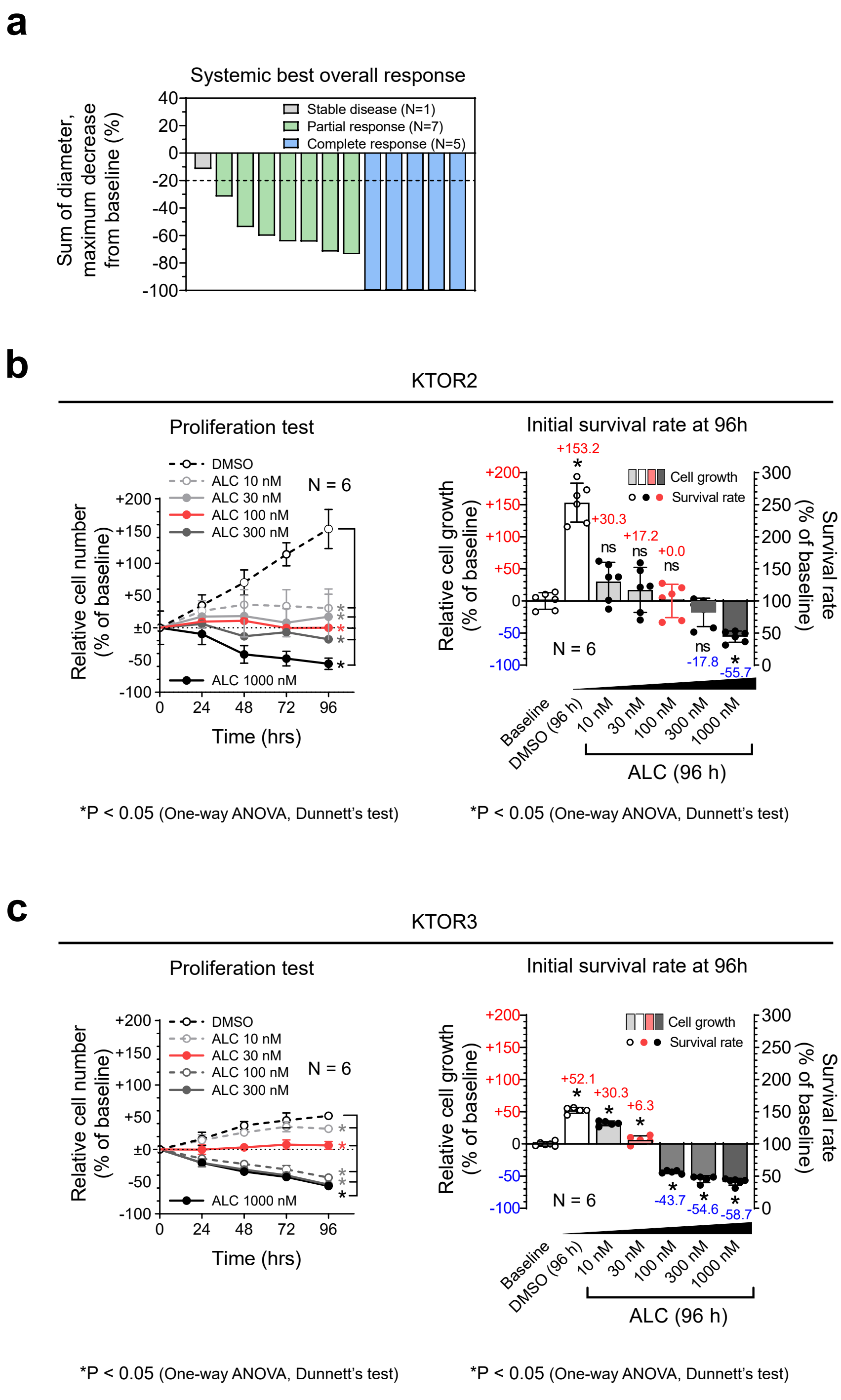

Supplementary Figure 1

a) A waterfall plot of the changes in tumor diameter based on RECIST guideline in the 13 patients (Supplementary Table S1) with ALK-rearranged lung cancer treated with alectinib (ALC). b-c) ALK-rearranged cells may survive, but their proliferation *in vitro* was significantly inhibited by ALC. Proliferation tests and the initial survival rates of KTOR2 (b) and KTOR3 (c) cells exposed to stepwise increases in the concentrations of ALC or to dimethylsulfoxide (DMSO). The significance of differences from the DMSO treatment was calculated using Dunnett's multiple comparison test. Error bars indicate ±S.D. * $P < 0.05$.

Supplementary Figure 2

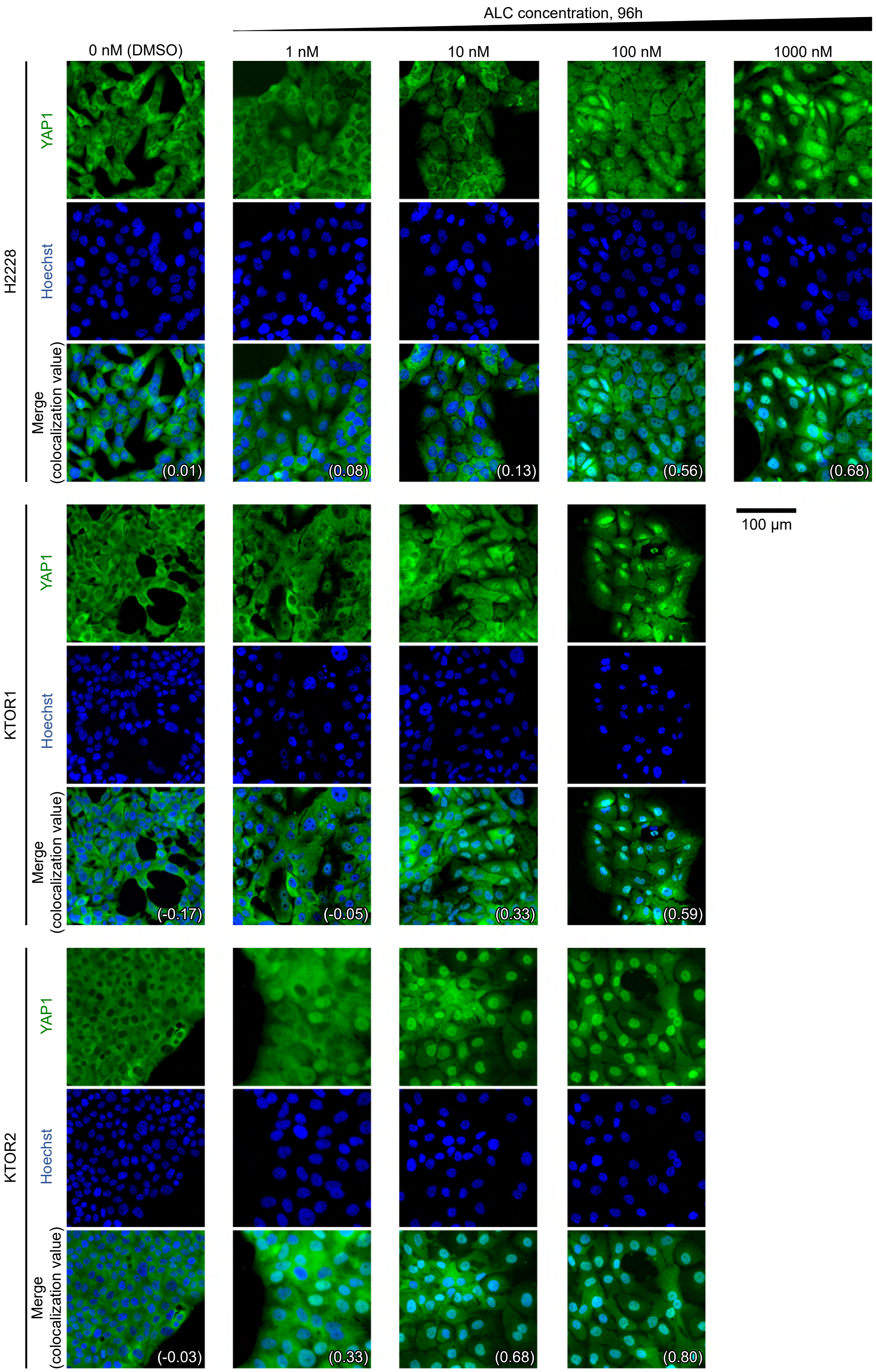

Supplementary Figure 2: The cell area was increased and YAP1 was activated by *in vitro* treatment with anaplastic lymphoma kinase (ALK) inhibitors

The cell adhesion area was increased and YAP1 was localized to the nucleus by exposure of the ALK-rearranged lung cancer cell lines H2228, KTOR1, and KTOR2 to alectinib (ALC).

Supplementary Figure 3

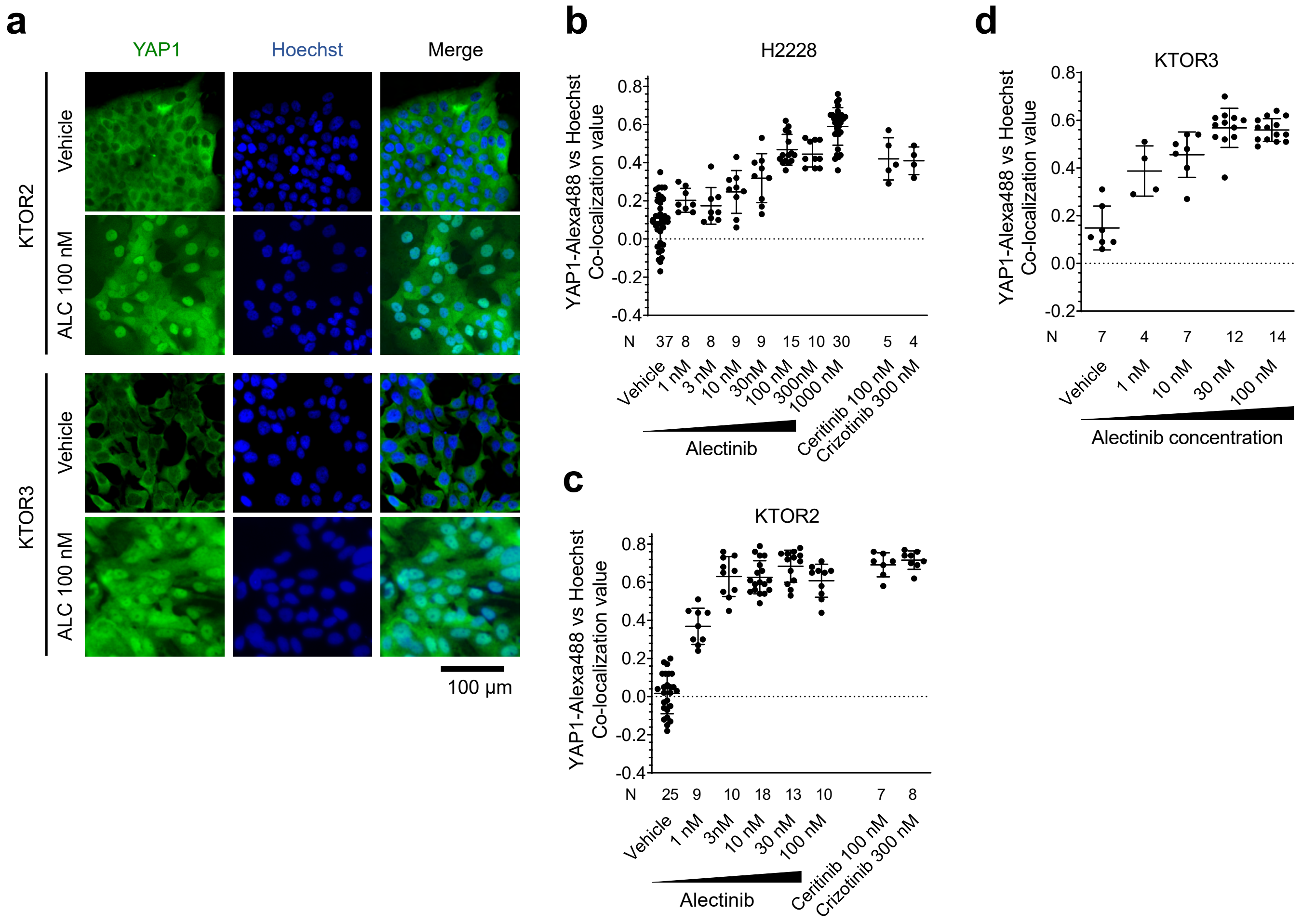

Supplementary Figure 3: YAP1 was activated by ALK inhibitors *in vitro*

a) YAP1 localized in the nucleus when anaplastic lymphoma kinase (ALK)-rearranged lung cancer cells, KTOR2 and KTOR3, were exposed to alectinib (ALC). b-d) Dose dependency of the nuclear localization of YAP1 in H2228, KTOR2, and KTOR3 cells. Error bars indicate ±S.D.

Supplementary Figure 4

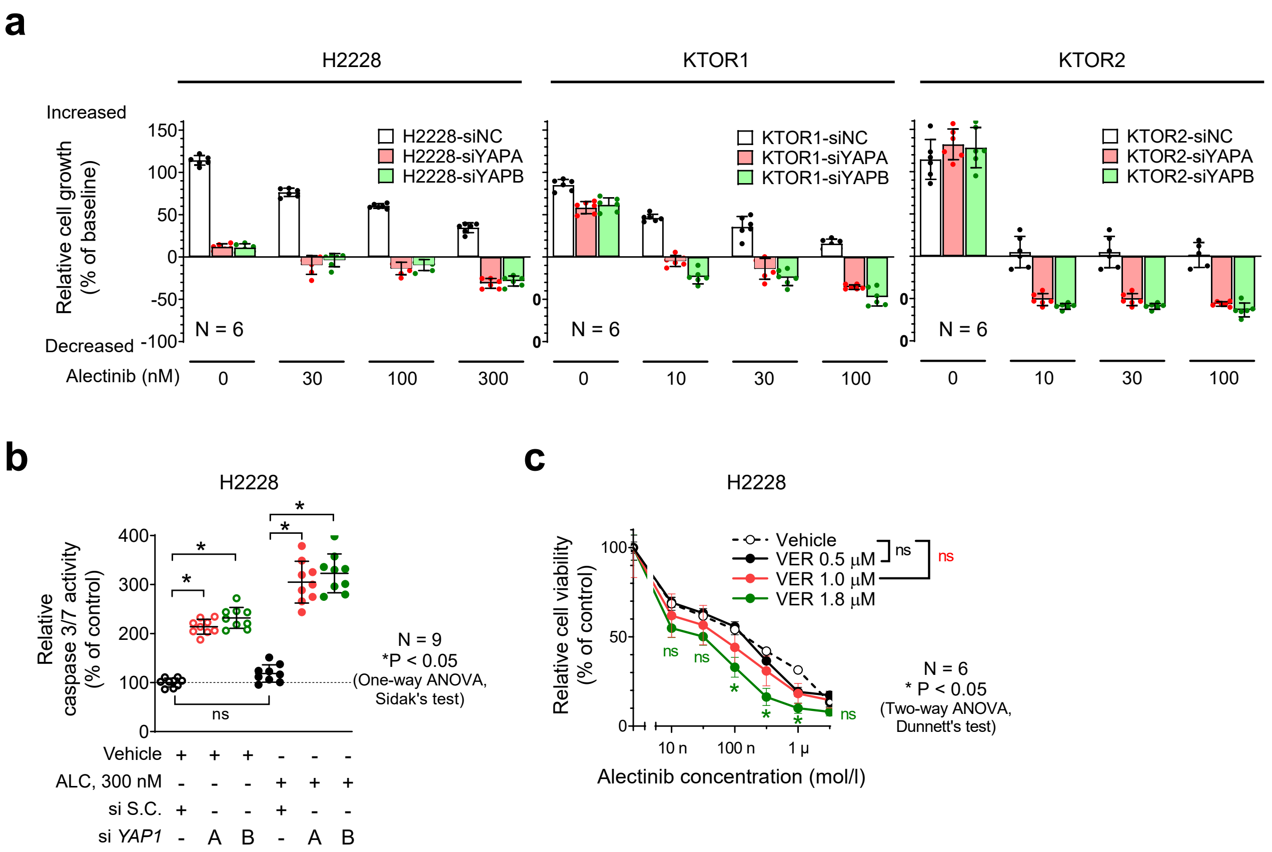

Supplementary Figure 4: YAP1 inhibition induced apoptosis and suppressed initial survival in ALK-rearranged cells

a) Complete results on the initial survival rates of anaplastic lymphoma kinase (ALK)-rearranged cells following knockdown of YAP1 and exposure to alectinib (ALC) or vehicle. b) Apoptosis assay using Caspase-Glo® in H2228 cells exposed to ALC or vehicle in combination with the knockdown of YAP1. Significance was evaluated using a one-way ANOVA followed by Sidak's multiple comparison test. c) Cell viability of H2228 cells exposed to ALC in the presence of verteporfin (VER). Significance was evaluated using a two-way ANOVA followed by Dunnett's multiple comparison test. Error bars indicate ±S.D.

Supplementary Figure 5

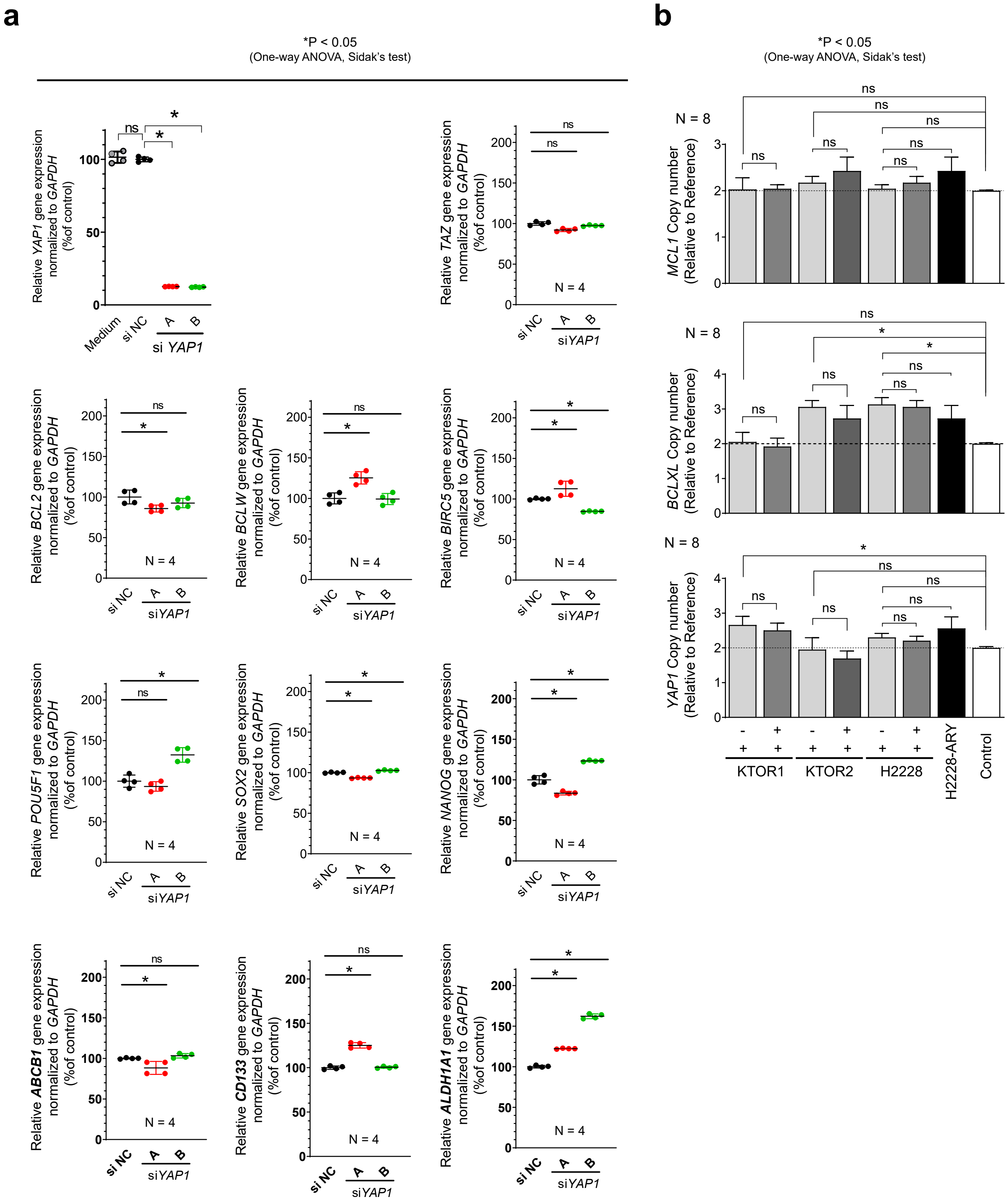

Supplementary Figure 5: qRT-PCR screening for effectors of YAP1 for initial survival of the ALC-resistant ALK-rearranged cell line, H2228-ARY.

a) The individual values for gene expression evaluated by qRT-PCR when YAP1 gene expression in H2228ARY was inhibited using siRNA. Heatmap data are shown in Fig. 5g. Significance was evaluated using a one-way ANOVA followed by Sidak's test. b) Gene amplification analysis on H2228, KTOR1, KTOR2, and H2228-ARY. H2228, KTOR1, and KTOR2 cells were evaluated when these cells were exposed to DMSO or alectinib (ALC). Significance was evaluated using a one-way ANOVA followed by Sidak's test. Error bars indicate ±S.D.

Supplementary Figure 6

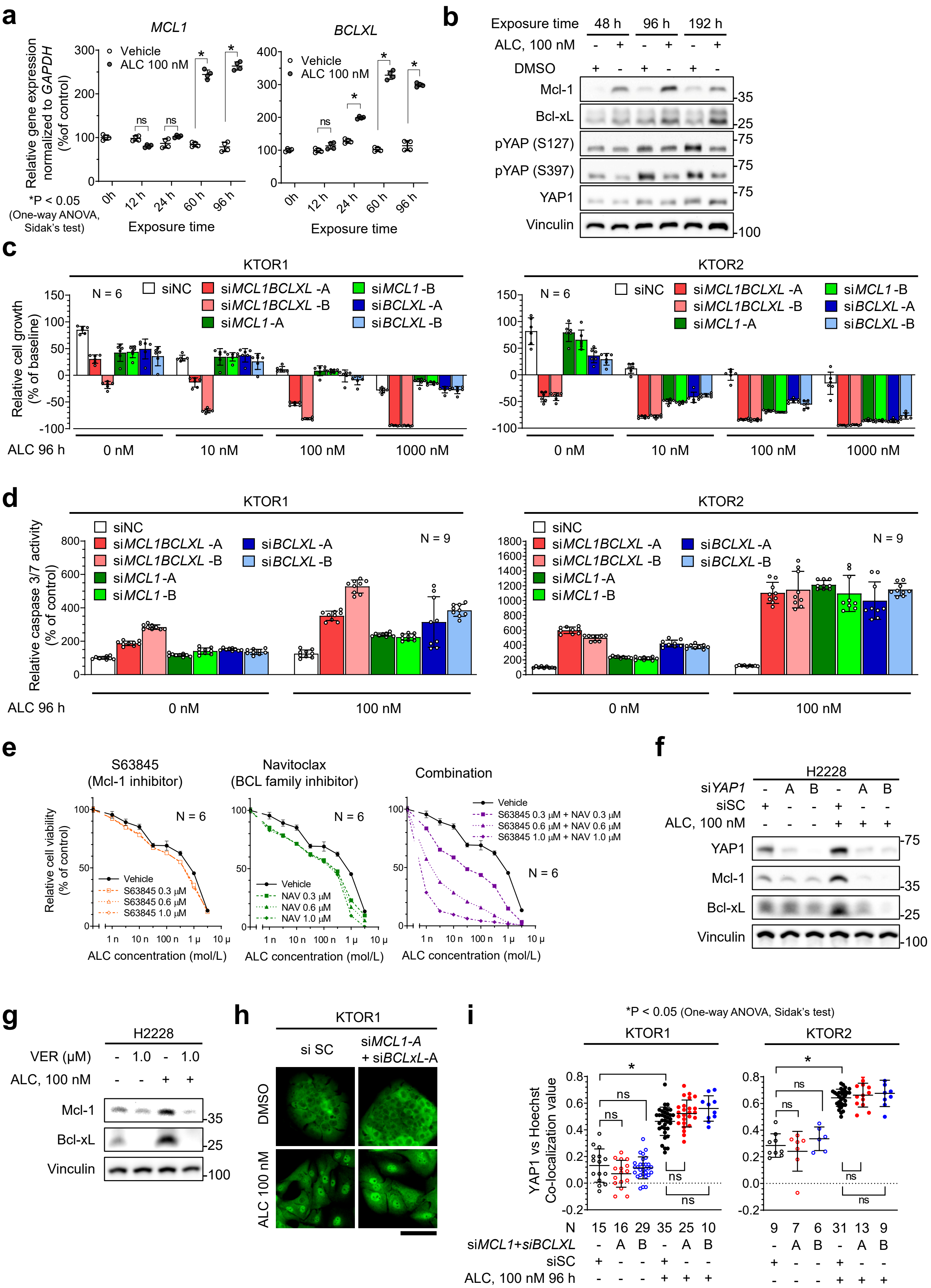

Supplementary Figure 6: Increases in Mcl-1 and Bcl-xL expression were mediated by YAP1

a-b) ALC-mediated time dependency of the expression of *MCL1* and *BCLXL* in KTOR1 detected by qRT-PCR (a) and immunoblotting (b). a) Gene expression values are normalized to GAPDH and relative expression to 0 h was plotted. Significance was evaluated using a one-way ANOVA followed by Sidak's test. b) Evaluation of Mcl-1 expression, Bcl-xL expression, and YAP phosphorylation. Cell lysates were immunoblotted with the indicated antibody. c,d) Initial survival rates (c) and caspase 3/7 activities (d) of KTOR1 and KTOR2 when cells were exposed to alectinib (ALC) or vehicle in combination with the knockdown of *MCL1* and *BCLXL*. Summarized results are shown in Fig. 6c, d. e) Pharmacological inhibition of both Mcl-1 and Bcl-xL increased ALC sensitivity in KTOR1. Cell viability assays on KTOR1 cells to ALC in the presence of an Mcl-1 inhibitor (S63845, left), Bcl-xL inhibitor (Navitoclax, middle), or their combination (right) are shown. f-g) Increases in Mcl-1 and Bcl-xL expression were cancelled by the inhibition of YAP1 in H2228 cells. YAP1 activity was inhibited using siRNA (f) or verteporfin (g). h) YAP1 distribution in KTOR1 and KTOR2 cells with the knockdown of *MCL1* and *BCLXL*. Scale bar = 100μm. i) The nuclear localization of YAP1 was not altered by the knockdown of *MCL1* and *BCLXL*. Significance was evaluated using a one-way ANOVA followed by Sidak's test.

ALC: alectinib, DMSO: dimethyl sulfoxide, VER: verteporfin, siSC: si scrambled control. Error bars indicate ±S.D.

Supplementary Figure 7

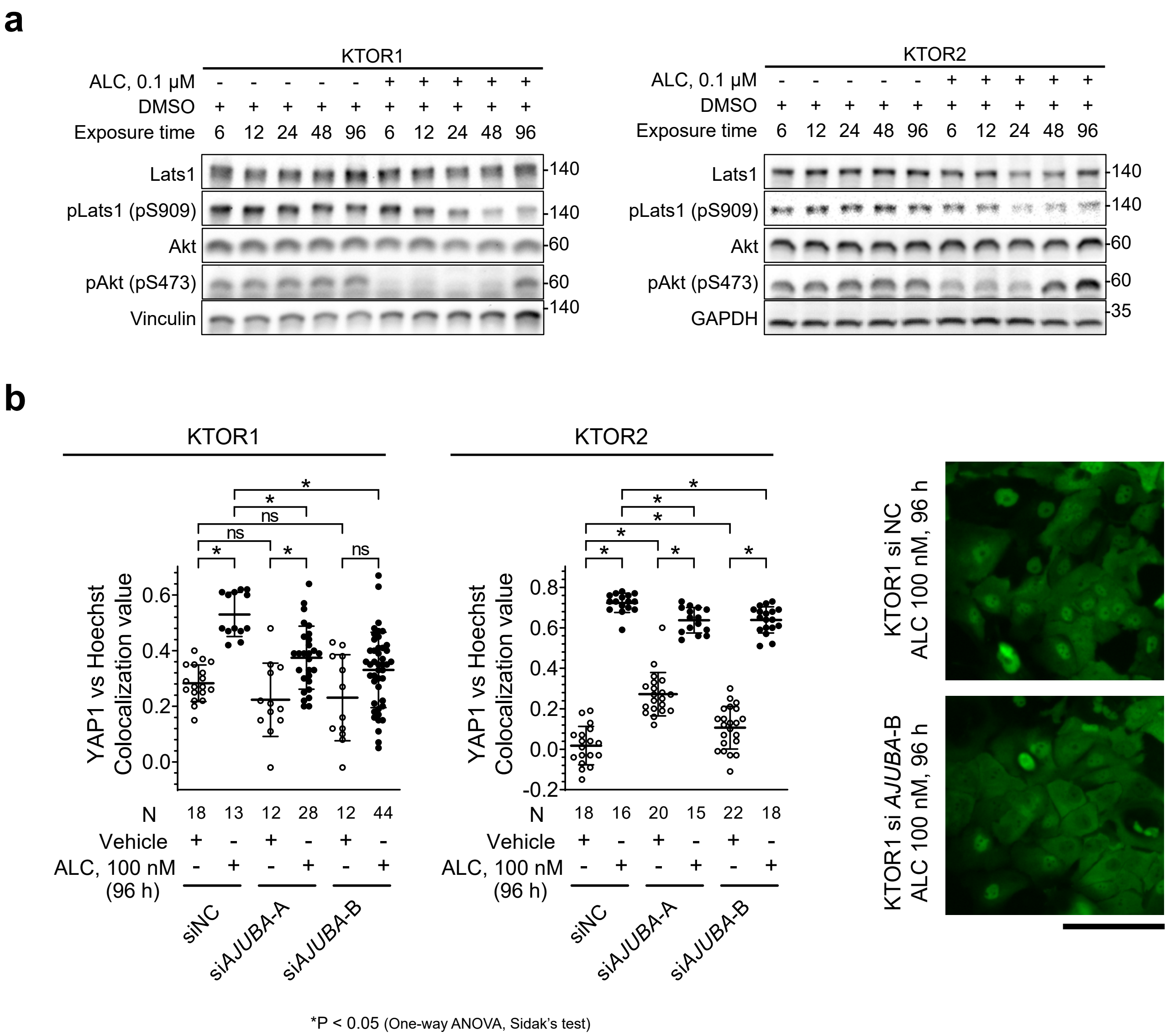

Supplementary Figure 7: YAP1 activation may be regulated by multiple factors

a) Alectinib (ALC)-mediated time dependency of the phosphorylation of Lats1 and Akt in KTOR1 and KTOR2 detected by immunoblotting. b) YAP1 distribution in ALK-rearranged cells with the knockdown of *AJUBA*. The colocalization value was significantly decreased when AJUBA was suppressed by ALC exposure. Significance was evaluated using a one-way ANOVA followed by Sidak's test. Error bars indicate ±S.D. Scale bar = 100µm.

Supplementary Figure 8

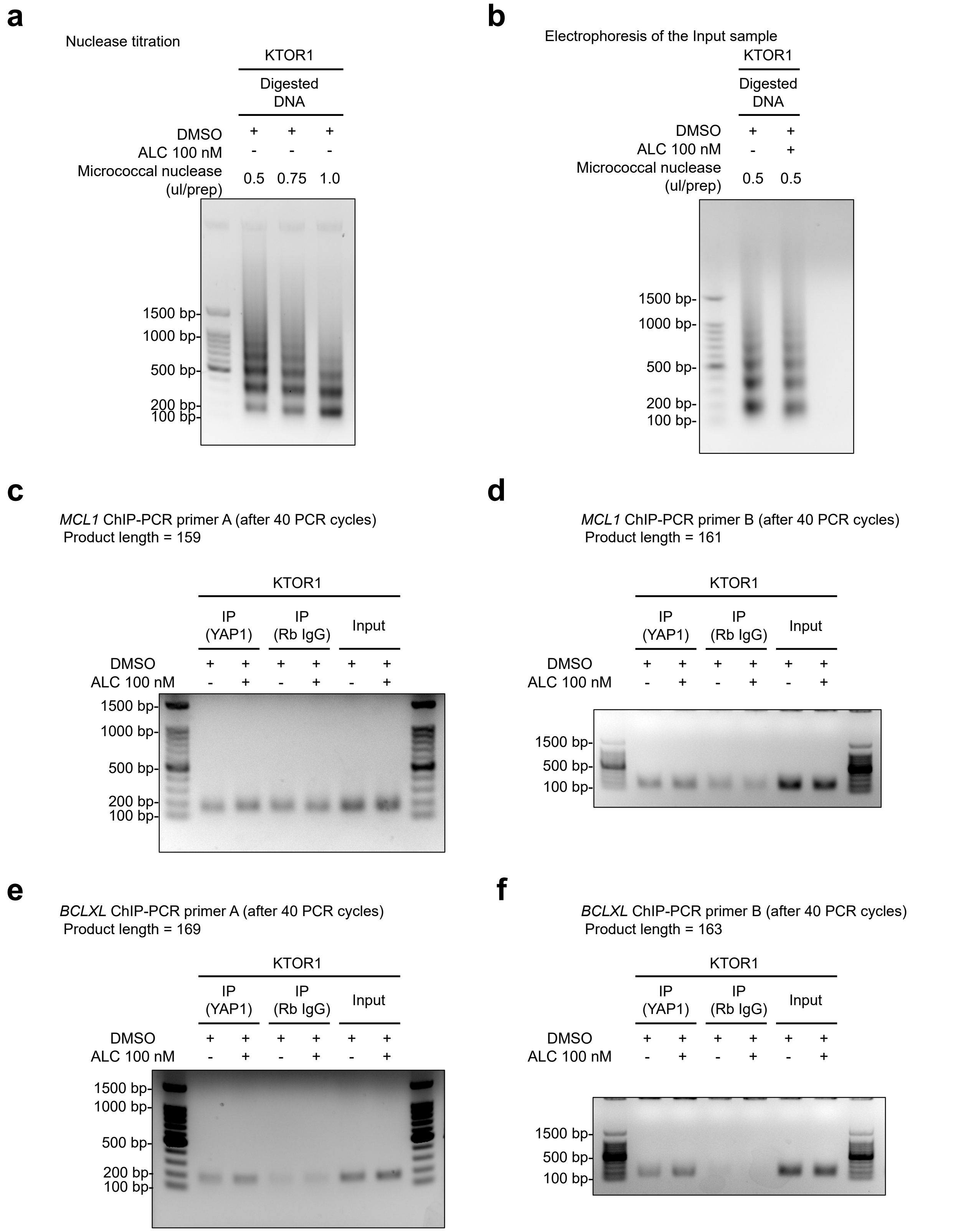

Supplementary Figure 8: Supplementary information on the ChIP-qPCR assay

a) Chromatin immunoprecipitation (ChIP) was performed using an enzymatic method. Micrococcal nuclease (Cell Signaling Technology, #10011) was titrated to identify the optimal nuclease dose for KTOR1. Electrophoresis of digested DNA samples indicated that 0.5–1.0 µl of nuclease per prep was appropriate for ChIP assays. b) Electrophoresis of digested cross-linked DNA input samples used for this assay. The extent of DNA digestion in ALC-treated KTOR1 was similar to that in DMSO-treated KTOR1. c-f) Electrophoresis of immunoprecipitated or input DNA samples amplified after 40 PCR cycles. All four primer pairs specifically amplified the targeted PCR products. Quantified data are shown in Fig. 6h.

ALC: alectinib, DMSO: dimethyl sulfoxide

•

Supplementary Figure 9

Uncropped blots in Figure 5h

Bcl-xL (KTOR1) (30kDa)

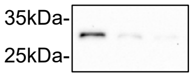

Cleaved PARP (89kDa)

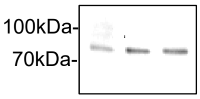

Mcl-1 (KTOR1) (40kDa)

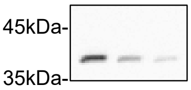

YAP1 (KTOR1) (65kDa)

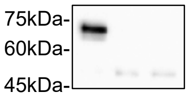

Vinculin (KTOR1) (110kDa)

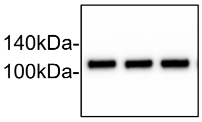

Uncropped blots in Figure 6b

EML4-ALK
(variant 1: 120 kDa, variant 3: 90 kDa)

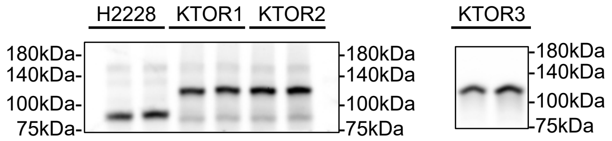

Lats1 (140 kDa)

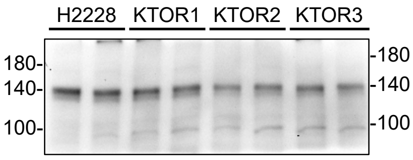

EML4-pALK
(variant 1: 120 kDa, variant 3: 90 kDa)

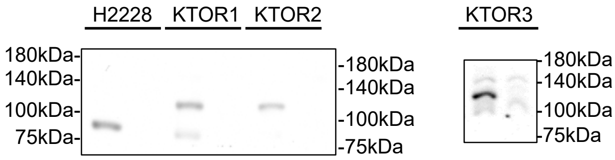

Phospho-Lats1 (S909) (140 kDa)

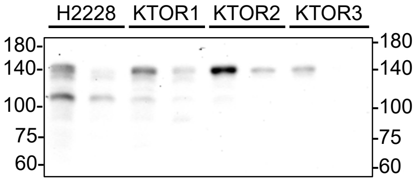

YAP1 (65-75 kDa)

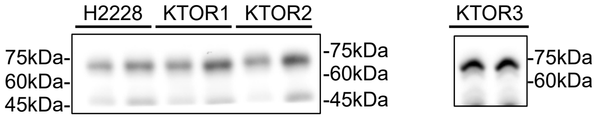

Akt (60 kDa)

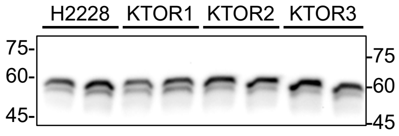

Phospho-YAP1 (S127) (65-75 kDa)

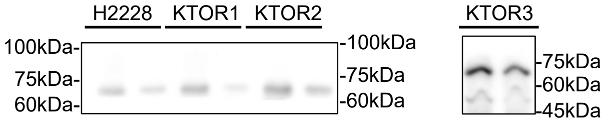

Phospho-Akt (S473) (60 kDa)

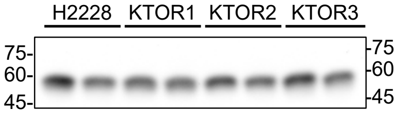

Mcl-1 (40kDa)

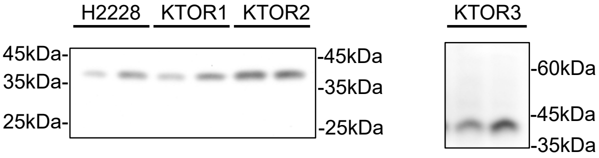

Vinculin (110kDa)

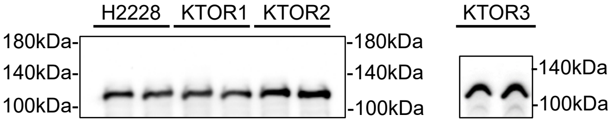

Bcl-xL (30kDa)

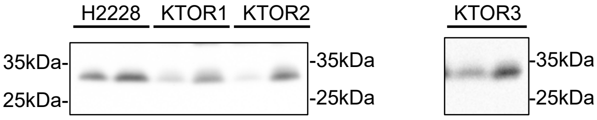

Uncropped blots in Figure 6e

Bcl-xL (KTOR1) (30kDa)

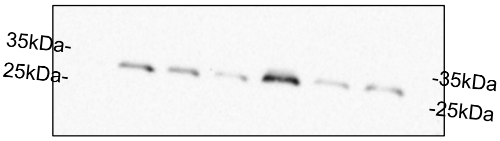

Bcl-xL (KTOR2) (40kDa)

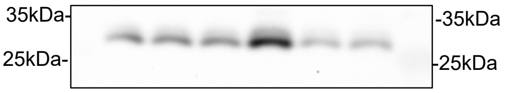

YAP1 (KTOR1) (65kDa)

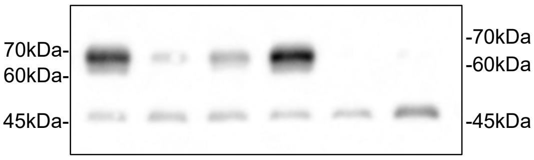

YAP1 (KTOR2) (65kDa)

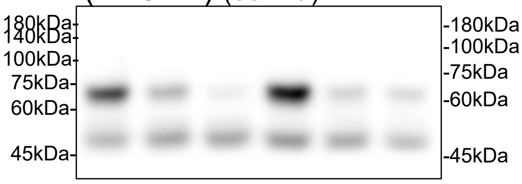

Mcl-1 (KTOR1) (40kDa)

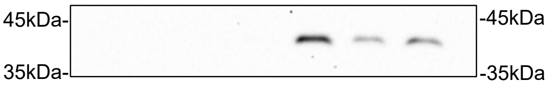

Mcl-1 (KTOR2) (40kDa)

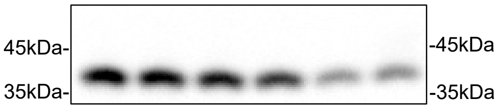

Vinculin (KTOR1) (110kDa)

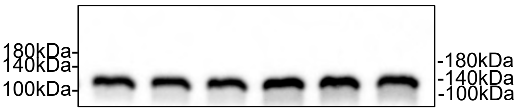

Vinculin (KTOR2) (110kDa)

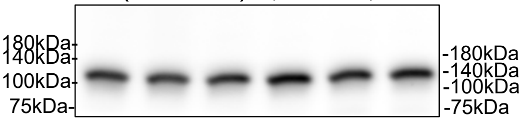

Uncropped blots in Figure 6f

Mcl-1 (40kDa)

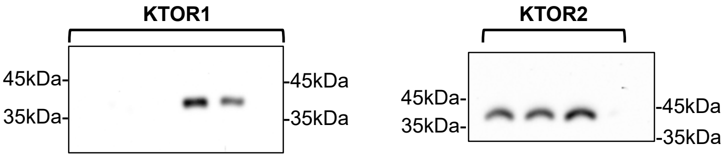

Bcl-xL (30kDa)

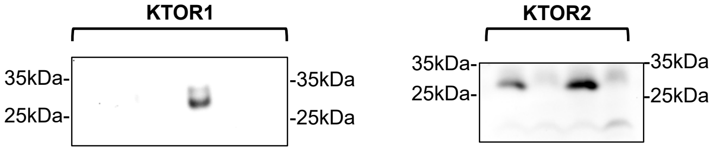

Vinculin (110kDa)

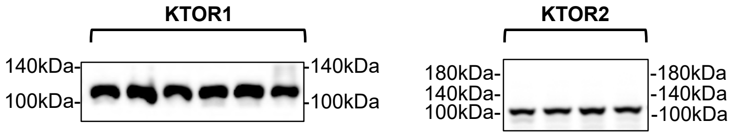

Uncropped blots in Figure 7a

EML4-ALK (Variant 3: 90kDa)

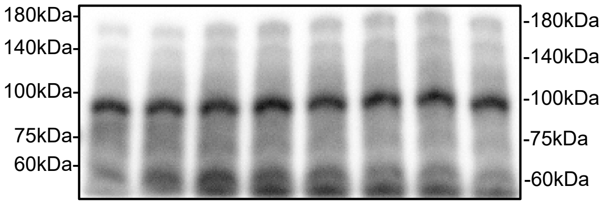

EML4-ALK (Variant 1: 110kDa)

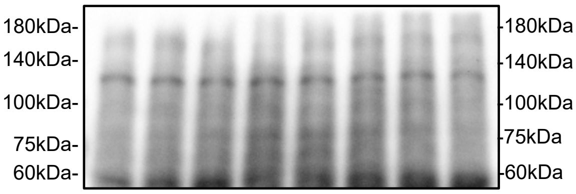

Phospho-EML4-ALK (Y1604)
(H2228 xenograft; Variant 3: 90kDa)

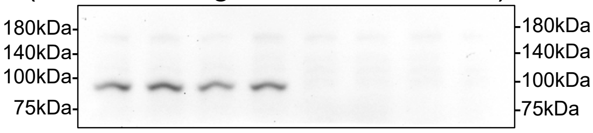

Phospho-EML4-ALK (Y1604)
(KTOR1 xenograft; Variant 1: 110kDa)

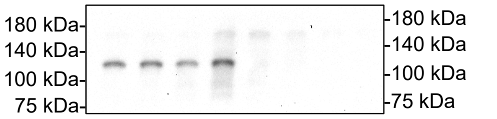

Mcl-1 (H2228 xenograft) (40kDa)

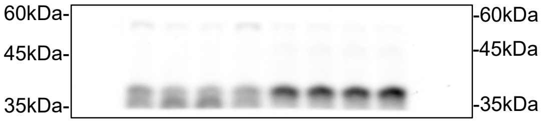

Mcl-1 (KTOR1 xenograft) (40kDa)

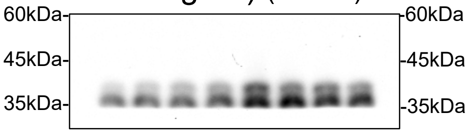

Bcl-xL (H2228 xenograft) (30kDa)

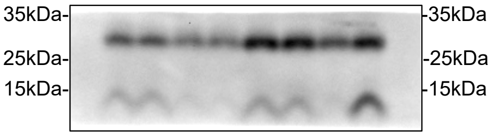

Bcl-xL (KTOR1 xenograft) (40kDa)

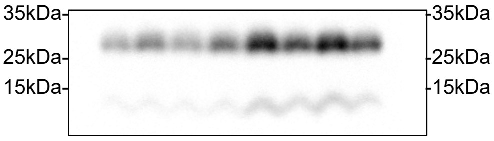

Vinculin (H2228 xenograft) (110kDa)

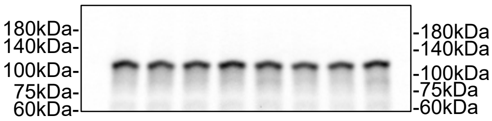

Vinculin (KTOR1 xenograft) (110kDa)

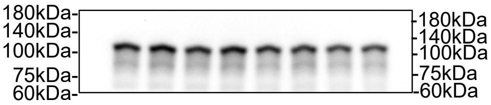

Uncropped blots in Figure 7b

H2228

Mcl-1 (40 kDa)

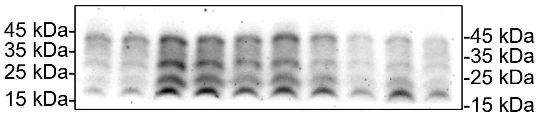

Bcl-xL (30 kDa)

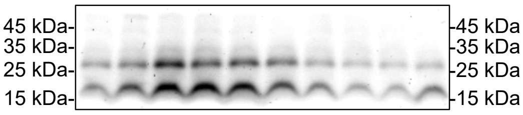

Cleaved Caspase (Asp175) (17,19 kDa)

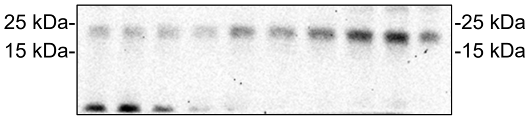

Vinculin (110kDa)

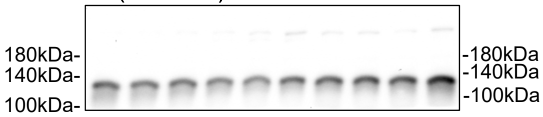

KTOR1

Mcl-1 (40 kDa)

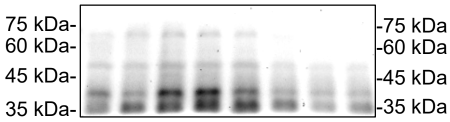

Bcl-xL (30 kDa)

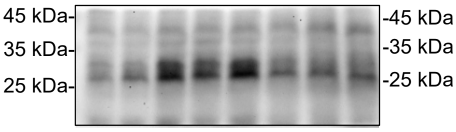

Cleaved Caspase (Asp175) (17,19 kDa)

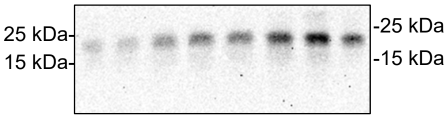

Vinculin (110kDa)

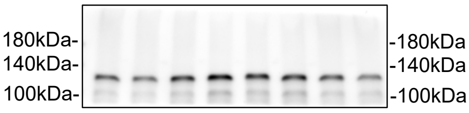

Supplementary Methods

Exome Capture and Sequencing

Each of 200 ng of genomic DNA obtained from the H2228 cells was fragmented with NEBNext dsDNA Fragmentase (New England Biolabs, MA) and purified using Agencourt AMPure XP beads (Beckman Coulter Inc., Brea, CA). The fragmented DNA were subjected to an exome capture procedure using a combination of SureSelect Human All Exon Kit V6 (target size: 60.5 mega-bases [Mb]) and SureSelect XT Low Input Reagents (Agilent Technologies, Santa Clara, CA) according to the manufacturer's protocols. The quality of the DNA libraries was assessed with an Agilent 2200 TapeStation High Sensitivity D1000 (Agilent Technologies, Santa Clara, CA). The pooled libraries of the samples were sequenced using the illumina HiSeq 1500 system (Illumina, Inc., San Diego, CA) in 101-base-pair (bp) paired-end reads.

Alignment and Variant Calling

The result of sequence reads were aligned to the human reference genome (GRCh37/hg19 + decoy sequences) obtained from the 1000 Genomes FTP site (ftp://ftp.1000genomes.ebi.ac.uk/) using the Burrows-Wheeler Aligner (BWA) version 0.7.151. Multiple identical reads from the exact same fragment were marked as duplicates and removed using Picard Tools version 1.119 (http://picard.sourceforge.net.). Local realignment around known indels and base quality score

recalibration were performed with the Genome Analysis Toolkit (GATK) 2-Lite version 2.3-9 (https://www.broadinstitute.org/gatk/). We used two methods of variant calling: UnifiedGenotyper of GATK and FreeBayes version 0.9.15. Parameters of GATK for filtering variants were determined as recommended by the GATK best practice guide (https://www.broadinstitute.org/gatk/). Variants called by FreeBayes were filtered out less than phred quality score 20. The consensus of two primary call sets from the different variant callers was used for the further analysis.

Variant Annotation and Filtering

Functional annotations of the Ensembl database GRCh37.752 and the possible effects of variants were added using SnpEff version 4.0E3. Using these annotations, the variants were filtered first for those that were predicted to alter amino acid sequences (missense, nonsense, and splice-site mutations and indels in coding regions), and then for those that were rare (<1.0% Minor Allele Frequencies [MAF] in the HapMap-JPT [Japanese in Tokyo, Japan], the 1000 Genomes EAS [the East Asian population including 104 Japanese individuals]4, or the Human Genetic Variation Database [HGVD] (http://www.genome.med.kyoto-u.ac.jp/SnpDB/), which contained genetic variations determined by WES in 1,208 Japanese individuals.

Supplementary References

1 Li, H. & Durbin, R. Fast and accurate short read alignment with Burrows-Wheeler transform. Bioinformatics 25, 1754-1760 (2009).

2 Flicek, P. et al. Ensembl 2014. Nucleic Acids Res. 42, D749-755 (2014).

3 Cingolani, P. et al. A program for annotating and predicting the effects of single nucleotide polymorphisms, SnpEff: SNPs in the genome of Drosophila melanogaster strain w1118; iso-2; iso-3. Fly 6, 80-92 (2012).

4 Abecasis, G. R. et al. An integrated map of genetic variation from 1,092 human genomes. Nature 491, 56-65 (2012).
