# Supplementary material for: YAP1 mediates survival of ALK-rearranged lung cancer cells treated with alectinib via pro-apoptotic protein regulation
Source: Nat Commun. 2020 Jan 3;11:74. doi: 10.1038/s41467-019-13771-5 (PMC6941996; doi:10.1038/s41467-019-13771-5)
Supplement: Supplementary file 2 — Description of Additional Supplementary Files [file 41467_2019_13771_MOESM2_ESM.docx]

Description of Additional Supplementary Files

**Supplementary Data 1:** The whole result of proteome analysis

**Supplementary Data 2:** Gene ontology (GO) analysis and KEGG pathway analysis on proteome

**Supplementary Data 3:** Results of whole exome sequencing on H2228ARY and H2228
