# Supplementary material for: YAP1 mediates survival of ALK-rearranged lung cancer cells treated with alectinib via pro-apoptotic protein regulation
Source: Nat Commun. 2020 Jan 3;11:74. doi: 10.1038/s41467-019-13771-5 (PMC6941996; doi:10.1038/s41467-019-13771-5)
Supplement: Supplementary file 7 — Reporting Summary [file 41467_2019_13771_MOESM7_ESM.pdf]

nature research

Corresponding author(s): Hiroaki OZASA

Last updated by author(s): Nov 3, 2019

Reporting Summary

Nature Research wishes to improve the reproducibility of the work that we publish. This form provides structure for consistency and transparency in reporting. For further information on Nature Research policies, see Authors & Referees and the Editorial Policy Checklist.

Statistics

For all statistical analyses, confirm that the following items are present in the figure legend, table legend, main text, or Methods section.

n/a	Confirmed	
☐	☒	The exact sample size (*n*) for each experimental group/condition, given as a discrete number and unit of measurement
☐	☒	A statement on whether measurements were taken from distinct samples or whether the same sample was measured repeatedly
☐	☒	The statistical test(s) used AND whether they are one- or two-sided<br>*Only common tests should be described solely by name; describe more complex techniques in the Methods section.*
☒	☐	A description of all covariates tested
☐	☒	A description of any assumptions or corrections, such as tests of normality and adjustment for multiple comparisons
☐	☒	A full description of the statistical parameters including central tendency (e.g. means) or other basic estimates (e.g. regression coefficient) AND variation (e.g. standard deviation) or associated estimates of uncertainty (e.g. confidence intervals)
☐	☒	For null hypothesis testing, the test statistic (e.g. *F*, *t*, *r*) with confidence intervals, effect sizes, degrees of freedom and *P* value noted<br>*Give P values as exact values whenever suitable.*
☒	☐	For Bayesian analysis, information on the choice of priors and Markov chain Monte Carlo settings
☒	☐	For hierarchical and complex designs, identification of the appropriate level for tests and full reporting of outcomes
☒	☐	Estimates of effect sizes (e.g. Cohen's *d*, Pearson's *r*), indicating how they were calculated

Our web collection on statistics for biologists contains articles on many of the points above.

Software and code

Policy information about availability of computer code

Data collection	Thermo Tune Plus (Thermo Fisher Scientific, Waltham, MA, United States)<br>Xcalibur 2.1 (Thermo Fisher Scientific, Waltham, MA, United States)<br>QuantityOne v4.6 (Bio-Lad, Hercules, CA, United States)<br>Applied Biosystems 7300 Real-Time PCR System, SDS v 1.4.1 (Thermo Fisher Scientific, Waltham, MA, United States)<br>PerkinElmer 2030 Workstation (PerkinElmer, Waltham, MA, United States)
Data analysis	Proteome Discoverer 2.1 (Thermo Fisher Scientific, Waltham, MA, United States)<br>GraphPad Prism 8 (GraphPad software, La Jolla, CA, United States)<br>JMP Pro version 12.0 (SAS Institute, Inc., Cary, NC, United States)<br>Microsoft Excel 2017 (Microsoft Corporation, Redmond, WA, United States)<br>ImageJ/FIJI and its "Coloc2" plugin: (https://fiji.sc/)<br>WebGestalt: (http://www.webgestalt.org/)<br>Picard Tools version 1.119 (http://picard.sourceforge.net.)<br>Genome Analysis Toolkit (GATK) 2-Lite version 2.3-9: (https://www.broadinstitute.org/gatk/).<br>SnpEff: (http://snpeff.sourceforge.net/)

For manuscripts utilizing custom algorithms or software that are central to the research but not yet described in published literature, software must be made available to editors/reviewers. We strongly encourage code deposition in a community repository (e.g. GitHub). See the Nature Research guidelines for submitting code & software for further information.

Data

Policy information about availability of data

All manuscripts must include a data availability statement. This statement should provide the following information, where applicable:

- Accession codes, unique identifiers, or web links for publicly available datasets
- A list of figures that have associated raw data
- A description of any restrictions on data availability

Raw data flies of proteome analysis were deposited to jPOST (https://repository.jpostdb.org/, ID: JPST000637 and PXD014783).
Other data and materials used for this study are available from the corresponding author.

Field-specific reporting

Please select the one below that is the best fit for your research. If you are not sure, read the appropriate sections before making your selection.

☒ Life sciences ☐ Behavioural & social sciences ☐ Ecological, evolutionary & environmental sciences

For a reference copy of the document with all sections, see nature.com/documents/nr-reporting-summary-flat.pdf

Life sciences study design

All studies must disclose on these points even when the disclosure is negative.

Sample size	This research mainly targets for experimental research. A total of five cell lines: three patient-derived ALK-rearranged cell lines, one commercially available ALK-rearranged cell line (H2228), and one alectinib-resistant cell line established from H2228.
Data exclusions	We included all Alectinib-sensitive ALK-rearranged cell lines for experiment. Patient-derived Alectinib-resistant cell lines were excluded, because the theme of this paper was initial survival of alectinib.<br>In addition, the KTOR3 cell line was excluded from proteome analysis and some biochemical analysis because of its slow growing speed.<br>KTOR2 and KTOR3 cell lines were excluded from xenograft studies because these cell lines did not form xenografts.
Replication	Cell viability assays, cell proliferation assays, qPCR, and apoptosis assays were performed independently at least 3 times with at least triplet wells. Immunoblot in vitro experiments were performed at least twice from the samples obtained and each sample was subjected to at least 2 blots to confirm the results obtained. In cell viability assay, and cell proliferation assay, at least 5 replicates per assay were analyzed.<br>In co-localization analysis, measurements were performed on at least 3 samples and at least 6 images in each sample.<br>Proteome analysis was performed once for each cell lines. (In total 3 analysis for 3 cell lines)<br>Assessment of Mcl-1 and Bcl-xL expression and YAP1 localiztion in xenograft was performed in 4 replicates.<br>We did xenograft treatment study only once, but in each study at least 6 replicates were analyzed.
Randomization	In xenograft study, mice were randomized into 4 treatment groups using random numer table.
Blinding	We did not blinded during data collection and analysis, because we have to check the study procedure and analysis methods were correctly done. However, we tried to reduce biases by sharing and discussing data among at least 3 independent scientists.

Reporting for specific materials, systems and methods

We require information from authors about some types of materials, experimental systems and methods used in many studies. Here, indicate whether each material, system or method listed is relevant to your study. If you are not sure if a list item applies to your research, read the appropriate section before selecting a response.

Materials & experimental systems

n/a	Involved in the study	
☐	☒	Antibodies
☐	☒	Eukaryotic cell lines
☒	☐	Palaeontology
☐	☒	Animals and other organisms
☐	☒	Human research participants
☒	☐	Clinical data

Methods

n/a	Involved in the study	
☒	☐	ChIP-seq
☒	☐	Flow cytometry
☒	☐	MRI-based neuroimaging

Antibodies

Antibodies used	anti-YAP1 antibody, Manufacture: Santa Cruz Biotechnology, Catalog No: sc-101199, Clone: 63.7, Lot: L-2717<br>anti-YAP1 antibody, Manufacture: Cell Signaling Technology, Catalog No: #14074, Clone: D8H1X, Lot: 4<br>anti-Phospho-YAP (Ser127) antibody, Manufacture: Cell Signaling Technology, Catalog No: #13008, Clone: D9W2I, Lot: 4

	anti-Mcl-1 antibody, Manufacture: Cell Signaling Technology, Catalog No: #5453, Clone: D35A5, Lot: 4<br>anti-GAPDH antibody, Manufacture: Thermofisher Scientific, Catalog No: MA5-15738, Clone: GA1R, Lot: SD246136<br>anti-Bcl-xL antibody, Manufacture: Cell Signaling Technology, Catalog No: #2764, Clone: 54H6, Lot: 6<br>anti-Cleaved PARP antibody, Manufacture: Cell Signaling Technology, Catalog No: #5625, Clone: D64E10, Lot: 13<br>anti-Vinculin antibody, Manufacture: Abcam, Catalog No: ab18058, Clone: SPM227, Lot: GR210315-1<br>anti-Phospho-ALK(Tyr1604) antibody, Manufacture: Cell Signaling Technology, Catalog No: #3341, Lot: 7<br>anti-ALK antibody, Manufacture: Cell Signaling Technology, Catalog No: #3333, Clone: C26G7, Lot: 7<br>Cleaved Caspase-3 (Asp175), Manufacture: Cell Signaling Technology, Catalog No: #9661, Lot: 23<br>Lats1, Manufacture: Cell Signaling Technology, Catalog No: #3477, Clone: C66B5, Lot: 7<br>Phospho-Lats1 (Ser909), Manufacture: Cell Signaling Technology, Catalog No: #9157, polyclonal, Lot: 2<br>Pan-Akt, Manufacture: Cell Signaling Technology, Catalog No: #4691, Clone: C67E7, Lot: 20<br>Phospho-Akt (Ser473), Manufacture: Cell Signaling Technology, Catalog No: #4060, Clone: D9E, Lot: 19
Validation	anti-YAP1 antibody (Clone: 63.7), Mouse monoclonal, Immunoblotting (IB) and immunohistochemistry (IHC)<br>anti-YAP1 antibody (Clone: D8H1X), Rabbit monoclonal, Chromatin immunoprecipitation (ChIP)<br>anti-Phospho-YAP (Ser127) antibody, Rabbit monoclonal, IB<br>anti-Mcl-1 antibody, Rabbit monoclonal, IB<br>anti-GAPDH antibody, Mouse monoclonal, IB<br>anti-Bcl-xL antibody, Rabbit monoclonal, IB<br>anti-Cleaved PARP antibody, Rabbit monoclonal, IB<br>anti-Vinculin antibody, Mouse monoclonal, IB<br>anti-Phospho-ALK(Tyr1604) antibody, Rabbit monoclonal, IB<br>anti-ALK antibody, Rabbit monoclonal, IB<br>anti-Cleaved Caspase-3 antibody, Rabbit polyclonal, IB<br>anti-Lats1, Rabbit monoclonal, IB<br>anti-Phospho-Lats1 (Ser909), Rabbit polyclonal, IB<br>anti-Pan-Akt, Rabbit monoclonal, IB<br>anti-Phospho-Akt (Ser473), Rabbit monoclonal, IB

Eukaryotic cell lines

Policy information about cell lines

Cell line source(s)	The NCI-H2228 (EML4-ALK variant 3a/b E6; A20) cell line was provided by the American Type Culture Collection in 2016. KTOR1, KTOR2, and KTOR3 cell lines were established in our institution at 2014-2017.
Authentication	Cell lines were authenticated by DNA fingerprinting. H2228 cells were authenticated when the cells were provided from ATCC.
Mycoplasma contamination	All cells were tested in 2018 for Mycoplasma using the MycoAlert™ Mycoplasma Detection Kit (Lonza, Basel, Switzerland).
Commonly misidentified lines<br>(See ICLAC register)	Commonly misidentified lines were not used in the study.

Animals and other organisms

Policy information about studies involving animals; ARRIVE guidelines recommended for reporting animal research

Laboratory animals	BALB/c-nu mice (CAnN.Cg-Foxn1nu/CrlCrlj) , female, 6-8 week of age<br>NSG mice (NOD.Cg-PrkdcscidIl2rgtm1Wjl/SzJ), female, 6-7 week of age
Wild animals	The study did not involve wild animals.
Field-collected samples	The study did not involve field-collected samples.
Ethics oversight	Animal experiments were approved by the institutional Animal Research Committee (ID: 17270)

Note that full information on the approval of the study protocol must also be provided in the manuscript.

Human research participants

Policy information about studies involving human research participants

Population characteristics	Patients with ALK-rearranged lung cancer who having been treated with alectinib.
Recruitment	The patients with alectinib-naive ALK-rearranged lung cancer were recruited from patients who regularly visited our institution.
Ethics oversight	The present study protocol was approved by the institutional Ethics Committee (certification number: R0996, G581).

Note that full information on the approval of the study protocol must also be provided in the manuscript.
